# MolPy: A Large Language Model-Friendly Toolkit for Reactive Topology Editing in Polymer Simulations

**DOI:** 10.1021/acs.jcim.6c01137

**Published:** 2026-07-01

**Authors:** Jichen Li, Fabian Schwarz, Wentao Guo, Ge Sun, Daniel Brandell

**Affiliations:** † Department of Chemistry-Ångström Laboratory, 8097 Uppsala University, 75121 Uppsala, Sweden; ‡ Division of Chemistry and Chemical Engineering, 6469 California Institute of Technology, Pasadena, California 91125, United States; § Courant Institute School of Mathematics, Computing, and Data Science, 5894 New York University, New York, New York 10012, United States; ∥ Department of Chemical and Biological Engineering, Tandon School of Engineering, New York University, Brooklyn, New York 11201, United States

## Abstract

Molecular modeling of synthetic polymers is today increasingly dependent on automation, high-throughput exploration, and the use of a diverse set of simulation tools. However, despite notable progress in molecular simulation methods, the practical infrastructure for constructing, modifying, and parametrizing polymer systems remains fragmented. Here, we launch a toolkit, MolPy, which aims to bridge this gap by providing an extensible and chemically intuitive platform for building polymer systems and orchestrating simulation workflows. The design philosophy emphasizes a clear separation of responsibilities, a stable and inspectable execution model, and a modular architecture that enables flexible extensions. In practice, the framework supports the introduction of new chemical building blocks and bonding schemes that automatically participate in construction, topology generation, and force-field assignment without requiring modification of existing modules. Interoperability with established simulation tools positions MolPy to support streamlined polymer modeling workflows that remain compatible with emerging large language model (LLM)-driven orchestration and other forms of automated pipeline construction.

## Graphical abstract

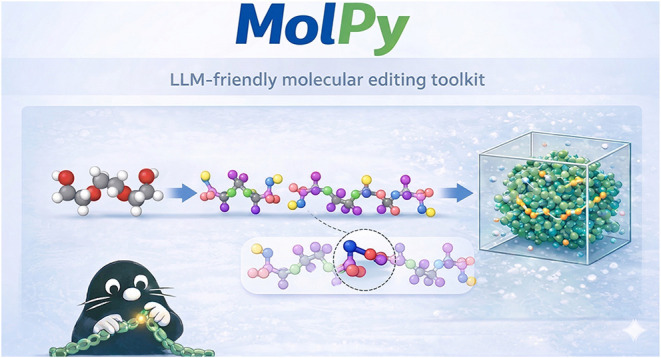

## Introduction

Molecular simulations are increasingly applied to not only rationalize known polymer systems but also to explore and design new materials.^1–3^ The relevant chemical space spans diverse monomer chemistries, functional-group patterns, and polymer architectures, including candidates proposed by generative models and inverse-design algorithms, as well as candidates considered in automated synthesis planning^4–6^ As this landscape grows, researchers require modeling tools that can accommodate new monomers, linkage rules, and topological items without reconstructing low-level infrastructure.^7^ A practical framework must therefore support composable methodologies and reuse established domain knowledge while keeping users from peripheral tasks such as file-format conversion, *ad hoc* data manipulation, and the coordination of heterogeneous external packages.^8^

A recent perspective by Turney and Matta has highlighted both the substantial progress and the persistent challenges in atomistic polymer modeling, with particular emphasis on polymer construction and parametrization workflows.^9^ In recent years, the field has advanced rapidly beyond earlier script-centric practices, and many contemporary tools now provide clearer APIs, improved documentation, and more reusable workflow components than were previously common. At the same time, practical modeling pipelines can still become fragmented when polymer growth, topology modification, force-field assignment, parametrization, and engine-specific input preparation must be coordinated across multiple software layers and file conversions.^10^ This has created growing interest in representations that are explicit, inspectable, and machine-readable, so that modeling logic can be composed, validated, and reused across tasks.^4,11^ The same requirement is further reinforced by recent advances in large language models (LLMs) and tool-integrated scientific agents, which rely on transparent operator semantics and well-structured interfaces rather than implicit procedural glue code.^12–14^ Establishing such modular and declarative abstractions is therefore increasingly important not only for workflow transparency and reproducibility, but also for enabling automated systems to interpret, adapt, and execute scientifically meaningful modeling procedures.

This development has been accompanied by a broad and rapidly growing software ecosystem spanning several layers of the polymer modeling. Polymer-specific builders and workflow platforms include SwiftPol,^15^ PSP,^16^ Polymatic,^17^ Polyply,^18^ PySoftK,^19^ HTPolyNet,^20^ CHARMM-GUI Polymer Builder,^21^ XPB,^22^ PolyConstruct,^23^ PEMD,^24^ RadonPy,^25^ and Chameleon.^26^ More general molecular assembly and system-preparation tools, such as mBuild,^7^ Moltemplate,^27^ and Packmol,^28^ remain important components of many practical pipelines. In parallel, topology and parametrization infrastructures, including the OpenFF^29^ polymer workflow and the MoSDeF/GMSO^30^ ecosystem, provide increasingly systematic ways to represent chemical information and assign force-field parameters, while newer learning-based approaches such as polyGen^31^ extend this landscape toward data-driven polymer structure generation. Taken together, these efforts show that polymer modeling is now an extremely active area with increasingly clear conceptual abstractions and well-documented algorithmic workflows.

Our group has worked on polymer and polyelectrolyte modeling for several years, including the development of the GroPoB^32^ protocol for automated construction. These efforts established a practical workflow for structure generation and simulation setup, and they proved to be effective for a range of routine modeling tasks. However, as the target problems became more complex, we increasingly encountered a recurring engineering bottleneck: chemically meaningful information had to be propagated consistently across graph editing, localized retypification, force-field manipulation, and downstream workflow generation through multiple transformation steps and software layers. This difficulty became particularly evident when workflows traversed heterogeneous representations, such as Atom in ASE,^33^ Mol in RDKit,^34^ and Struct in ParmEd,^35^ where interconversion could obscure assumptions, duplicate logic, and increase maintenance overhead.^35,36^ These recurring integration demands ultimately motivated the development of MolPy as a more unified and programmable molecular-modeling framework.

To address these challenges, we introduce MolPy, a modular framework for polymer molecular system construction and manipulation built from programmable components that can be composed according to the requirements of a given modeling task. MolPy separates molecular construction into decoupled, chemically meaningful modules, so tasks such as sequence definition, topology editing, parameter assignment, and system assembly can be carried out independently without obscuring their chemical meanings. For example, sequence generation from BigSMILES representations can be performed without subsequent atomistic construction, while topology editing and system preparation can be integrated into existing modeling workflows, as shown in Figure 1. MolPy represents molecular systems by using a consistent internal data model that is independent of simulation engines and file formats. This allows molecular operations to be applied directly to the system representation, while routine tasks such as connectivity updates, parameter assignments, and data conversions are handled in a uniform way.

**Figure 1:**
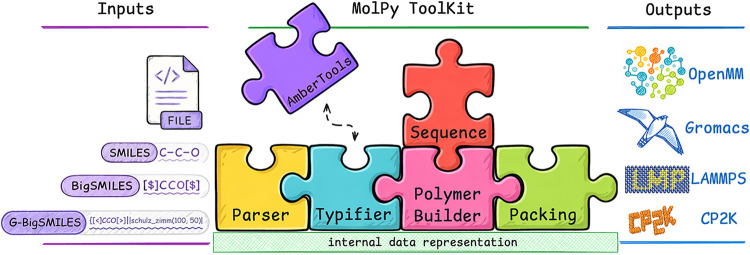
Overview of the MolPy toolkit. MolPy organizes molecular-modeling workflows as modular, reusable components built on a shared internal data representation. Diverse chemical inputs, including structure files, SMILES, BigSMILES, and G-BigSMILES, are transformed through core modules such as the parser, typifier, polymer builder, sequence handling, and packing engine, with optional integration of external tools such as AmberTools. This modular design enables flexible assembly of end-to-end workflows and export to multiple downstream simulation engines, including OpenMM,^37^ GROMACS,^38^ LAMMPS,^39^ and CP2K.^40^ Images and logos are reproduced in compliance with the corresponding licenses: OpenMM under the Apache License 2.0 or the project software licenses, GROMACS under the GNU Lesser General Public License v2.1 or later, LAMMPS under the GNU General Public License v2, and CP2K under the GNU General Public License v2 or later, with the CP2K logo used under the Creative Commons Attribution-ShareAlike 4.0 International License.^41–44^

Figure 2 presents the layered architecture of MolPy, in which clearly separated levels of abstraction, decoupled components, explicit type annotations, and well-defined interfaces together enable an LLM-friendly design that can support the robust modeling of complex polymer systems. MolPy is released as open-source software, with the full source code, documentation, and tutorials available at MolCrafts/molpy under the BSD 3-Clause license. The framework is under active development, with ongoing extensions and maintenance. The broader aim is to provide a flexible, community-driven platform that evolves with emerging research priorities and supports growing structural and chemical complexity, ultimately reducing the time required to move from the conceptual design to executable simulation.

**Figure 2:**
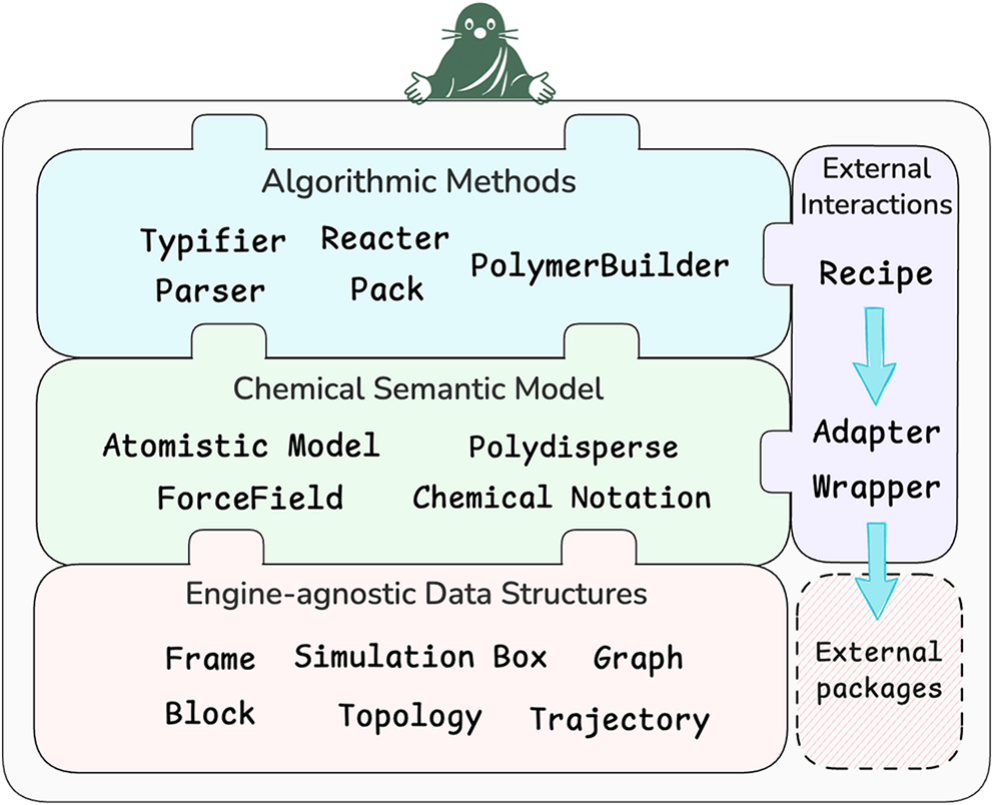
MolPy’s three-layer architecture comprises data structure layer, chemical semantics layer, and methods layer. The data structure layer provides the foundational representations used to describe geometry, topology, and simulation data. The chemical semantics layer specifies molecular models, force field definitions, and polymer descriptions. The methods layer implements higher-level operations—including typification, reactive linking, and spatial placement—and manages interoperability with external simulation packages, such as Packmol^28^ and RDKit.^34^

The remainder of this article is organized around MolPy’s architecture and its practical application. We begin by outlining the design principles that guide the framework and then introduce the core data structures that underpin its internal representation. These abstractions support a middle layer that captures chemical semantics and provides the foundation for a set of higher-level modules and methods. Together, these layers define a modular and extensible architecture for the polymer modeling. We illustrate the capabilities of this architecture through a sequence of four stepwise examples: Case I, manual monomer assembly; Case II, sequence-driven automatic polymer construction; Case III, chemistry-aware assembly via the REACTER protocol;^45^ and Case IV, stochastic polydisperse system generation based on G-BigSMILES.^46^

## Architecture and Methods

MolPy adopts a layered architecture that separates data representation, chemical semantics, and operational logic into components that can be tested and replaced independently. At its foundation, the framework provides explicit, engine-agnostic data structures, including column-based data tables, molecular graphs, and force-field parameter containers, that maintain a transparent and consistent internal state regardless of external file formats or simulation packages. The intermediate layer introduces domain-level data types such as all-atom and coarse-grained models, specific force-field and potential definitions Higher-level capabilities, including topology modification, parameter assignment, reactive linkage, and spatial arrangement, are implemented as operators and algorithms that transform these data types. External tools are integrated through minimal adapter interfaces that encapsulate engine-specific behavior. As illustrated in Figure 2, this architecture supports predictable and verifiable execution, enables automated orchestration, and preserves explicit, machine-interpretable semantics for each modeling operation.

MolPy represents molecular systems through a joint representation that combines two complementary views of structure. The first view is the Frame, which provides a static and numerically oriented layout that stores per-atom and topology-level quantities such as positions, velocities, charges, and type labels in columnar, index-aligned arrays. This representation follows the data models commonly used in molecular simulation engines and supports vectorized computation, efficient querying, and low-overhead data interchange. In parallel, MolPy maintains a molecular graph that records the system’s chemical connectivity. In contrast to the static nature of the Frame, the molecular graph is dynamic and supports adding, removing, and modifying atoms and bonds as required during topology editing and reactive updates. Both representations use the same atom identifiers, and neither representation requires a fixed attribute scheme. Atomic properties can be extended freely, and the Frame stores per-atom data in columns that can grow as needed. This flexibility allows the Frame and the molecular graph to be converted into each other while preserving a consistent description of the system. This joint representation enables a clear separation of responsibilities: the Frame provides an array-based, column-aligned representation optimized for efficient numerical operations on per-atom data, while the molecular graph supplies a chemically meaningful structure for editing and analysis. Together, they form the foundation for higher-level operations, such as topology modification, force-field assignment, and monomer assembly in MolPy.

MolPy introduces a dedicated Topology abstraction to support computations that depend solely on molecular connectivity rather than on atom-level attributes. This layer provides a clean interface for graph-based operations such as determining topological distances, locating neighboring atoms, identifying connected components, and extracting substructures. It also offers controlled procedures for adding, removing, or reassigning bonds when the connectivity changes. By separating connectivity data from the algorithms that operate on it, the topology layer supplies a uniform and reliable basis for querying, validating, and modifying polymer architectures. This abstraction enables higher-level capabilities including monomer linking, cross-link formation, reactive edits, and the automated generation of branched or networked polymer topologies.

MolPy defines a flexible ForceField abstraction that represents force field information independently of any specific simulation engine. The force field is expressed as a collection of Styles and Types. Style describes the mathematical form of bonded or nonbonded interactions, and Type stores the parameters associated with a particular structural motif. Both components inherit from a generic base class that stores topology-level metadata without assuming any specific functional form. Concrete subclasses, such as BondHarmonicStyle, together with their associated type definitions, specify the potential parameter schema and are responsible for generating the corresponding bond potential when exporting to a simulation engine. Typification is performed through graph-based queries on the topology, allowing interaction patterns to be matched to structural motifs rather than to positional or backend-specific assumptions. This design ensures that atom and topological item types can be reassigned consistently as bonds are created, removed, or modified during polymer growth or reactive edits. Because the ForceField abstraction is defined entirely in terms of molecular graph patterns and extensible style/type classes, new interaction forms or parametrizations can be introduced without altering the underlying infrastructure. Together, these features provide a stable and engine-agnostic semantic representation layer for the force field. They support downstream tasks such as topology validation, parameter assignment, and automated export of potentials to external simulation tools.

MolPy’s interoperability layer provides explicit and machine-interpretable interfaces to external tools, which makes multistage workflows easier to analyze, validate, and automate. These interfaces expose the intent of each external operation and clarify the data exchanged at every boundary, allowing both users and LLM-based agents to reason about complex procedures in a structured manner. To support this capability, MolPy separates external interactions into lightweight workflow utilities and two core interoperability components. At the workflow level, high-level utilities may package recurring multistep modeling procedures behind a single callable Python interface, with configuration specified at construction time and runtime inputs supplied at execution. These utilities are intended as convenience layers rather than low-level composable primitives. For procedures that depend on external software, Wrapper exposes third-party executables as Python-callable operations without embedding tool-specific assumptions into MolPy’s data model. Adapter provides bidirectional translation between MolPy’s internal representations and the data structures used by external packages, ensuring a predictable information flow in both directions, as shown in Figure 3. Together, this separation of responsibilities yields a reliable and extensible interoperability model in which external capabilities can be incorporated without compromising MolPy’s core abstractions. It also defines a stable interface for automation, supporting consistent construction and transformation of workflows across different stages of molecular modeling.

**Figure 3:**
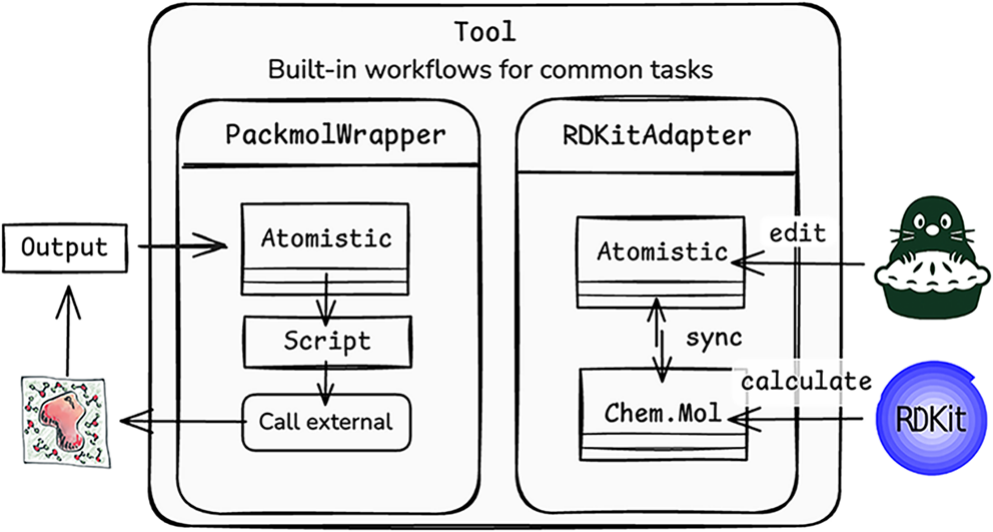
Schematic illustration of MolPy’s interoperability layer. The PackmolWrapper generates external scripts and invokes third-party tools to produce new atomistic configurations, whereas the RDKitAdapter synchronizes data between MolPy’s atomistic representation and RDKit’s Chem.Mol objects, supporting structure edits and property calculations. Together, these components enable complex workflows to be expressed in a declarative and reusable form. The Packmol and RDKit logos are reproduced in compliance with their respective open-source licenses: Packmol under the MIT License and RDKit under the BSD 3-Clause License. Where no separate logo-specific license is provided, the corresponding project license was followed.^47,48^

Building on its unified data model, MolPy provides a set of polymer construction modules that transform monomer-level specifications into simulation-ready atomistic chains. Parameter assignment in MolPy is handled at the Topology layer by Typifier. Its design is informed by both Foyer,^49^ which applies rule-based atom typing through SMARTS matching, and OpenForceField,^29^ which emphasizes chemical-pattern-based parameter assignment. Within MolPy, these ideas are adapted to a typed force-field representation because the Topology layer preserves explicit atom types, the current implementation performs parameter assignment through SMARTS-based matching of local chemical environments, followed by mapping to predefined types and corresponding force-field parameters. When no pattern matches, MolPy reports unmatched cases without implicit estimation, enabling users to extend rules or directly edit force-field definitions, while preserving transparency and reproducibility. While sharing the same SMARTS-based matching paradigm, MolPy adopts a different execution strategy by precompiling pattern dependencies, enabling more efficient and modular typing within the topology layer. Because typing is tied to structural motifs, bonded parameters remain consistent as users extend, rearrange, or modify chain connectivity. Reactive modifications are handled by the Reacter module, which applies user-defined rules to create or delete bonds, remove atoms, and update types while preserving topological validity. These chemically meaningful edits allow MolPy to express chain growth, functionalization, and cross-link formation directly on the molecular graph. Geometry-aware assembly is managed by a lightweight builder framework. A Placer component governs the spatial placement of monomers during chain construction, and a Connector component determines how monomers or functional groups are joined according to the registered Reacter rules. Together, they provide a consistent interface for generating linear, branched, sequence-defined, and networked polymer structures. These modules operate directly on typed frames and topologies, share a common interface, and can be composed into flexible workflows. The result is an engine-independent mechanism for converting high-level chemical descriptions into detailed, parametrized atomistic polymer models.

Across MolPy’s architecture, all construction, modification, and parametrization procedures are expressed as transformations over a shared molecular representation. This imposes a consistent boundary between structural semantics and downstream execution, ensuring that method-level components remain composable and mutually independent. By organizing polymer construction as a sequence of declarative, representation-level transformations, MolPy avoids entangling chemical logic with backend-specific concerns. As a result, complex polymer architectures can be assembled through method composition rather than task-specific control flow while preserving a clear separation between semantic intent and execution details.

## Case Studies

To illustrate how MolPy supports a wide range of polymer construction workflows, we present a sequence of use cases that progress from manual assembly toward increasingly automated and chemically informed procedures. These examples capture both traditional practices, in which polymer networks are created by joining prebuilt fragments, and more modern strategies that rely on sequence specifications, distribution-controlled chain ensembles, or reactive bond formation. By organizing the case studies along this trajectory, we highlight how MolPy preserves compatibility with established approaches while enabling modular and extensible methods for building atomistic polymer architectures. We examine four representative cases: (i) manual template-based construction similar to Moltemplate;^27^ (ii) deterministic assembly from user-defined monomer sequences; (iii) automated generation of chain-length distributions using G-BigSMILES^46^ definitions together with system size constraints; and (iv) the creation of REACTER^45^ protocol through MolPy’s topology and molecular-editing modules.

Building polymeric systems requires control over both covalent connectivity and spatial placement. Figure 4 shows a single bond-forming step in the construction of a poly­(ethylene oxide) (PEO) trimer. A bifunctional PEO monomer is first converted into a molecular object, expanded into a three-dimensional structure with RDKit, and assigned the corresponding bonded topology terms. A second monomer is then translated and rotated to align a compatible reactive site, after which the new bond is formed, the leaving atoms are removed, and the topology is regenerated and retyped. The figure shows only one growth step, corresponding to the transformation *P*_*n*_ + *M* → *P*_*n*+1_; repeated application of the same operation yields the full trimer.

**Figure 4:**
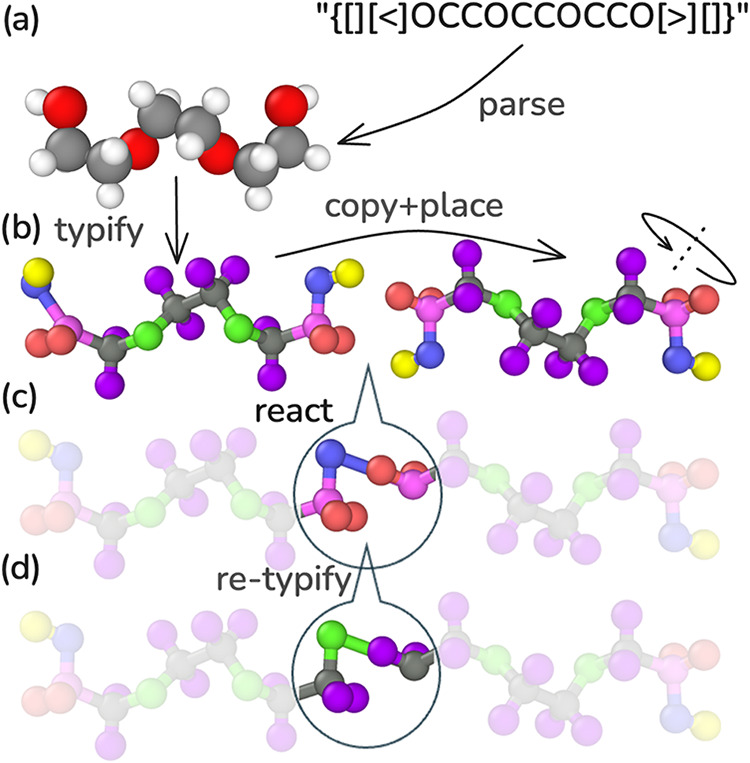
Schematic illustration of polymer construction in MolPy. (a) A textual repeat-unit representation is first parsed into a molecular building block, with atoms shown in standard element-based colors. (b) The building block is then typified and copied to generate the polymer sequence; this operation includes cloning the repeat unit, determining the placement of the next unit, and rotating it into a suitable orientation for connection. In panels (b–d), atoms are colored by their OPLS atom types. (c) The neighboring units are connected through a reaction step that modifies the local bonding pattern at the linking sites. (d) The connected structure is finally retypified so that atom types and associated force-field information are updated consistently with the new chemical environment.

This procedure is similar in spirit to template-based construction in Moltemplate,^27^ but is carried out here on a unified molecular representation rather than through engine-specific templates and scripts. Case II generalizes the same idea into an automated workflow: a Reacter applies bond-forming rules, a Connector selects the appropriate reaction pattern, and Placer and Orientator determine the monomer position and orientation. Together, these components extend the manual PEO growth step to a rule-driven workflow for larger and more complex polymer architectures.

Building on the Moltemplate-style construction demonstrated in case I, case II, in turn, illustrates how MolPy generalizes the same ideas into an automated and rule-driven construction workflow. Rather than manually stitching ports or positioning monomers, users can specify high-level chemical and architectural intent, and MolPy generates the corresponding atomistic structure through four coordinated components. The reactor identifies reactive sites, forms new bonds, and applies any required eliminations. A Connector manages a library of reactors and selects the appropriate one when linking different monomers. Placer determines the spatial arrangement by setting the orientation and position of monomers in the simulation box, thereby replacing the manual translation and rotation steps used in Case I. Together, these components transform the manual and geometry-driven workflow of Case I into an automated and chemically aware builder capable of producing consistent, sequence-controlled polymer architectures with minimal user effort.

A styrenic repeat unit together with bifunctional and trifunctional ethylene oxide building blocks are used here to illustrate the multilevel polymer representations supported in MolPy. We begin from atomistically resolved repeat-unit fragments with explicitly defined connection sites, as shown in Figure 5a–c, where the corresponding string-based descriptions encode a styrenic unit (STY), an EO2 unit with two connection sites, and an EO3 unit with three connection sites. These chemically defined building blocks are then lifted to a higher-level motif representation using abstract labels such as #STY, #EO2, and #EO3, from which more complex polymer architectures can be specified compactly. As illustrated in Figure 5d–f, this representation is not restricted to linear sequences, but can also describe branched, cyclic, and repeated graph-like topologies within the same declarative framework. In this way, MolPy separates local chemical definition from global architectural specification: the underlying chemistry is retained in the building-block motifs, while the connectivity between motifs is expressed through a CGSMILES-inspired graph formalism.^50^ The corresponding code example is shown in Figure 5, and the overall construction procedure is summarized in Algorithm 1.
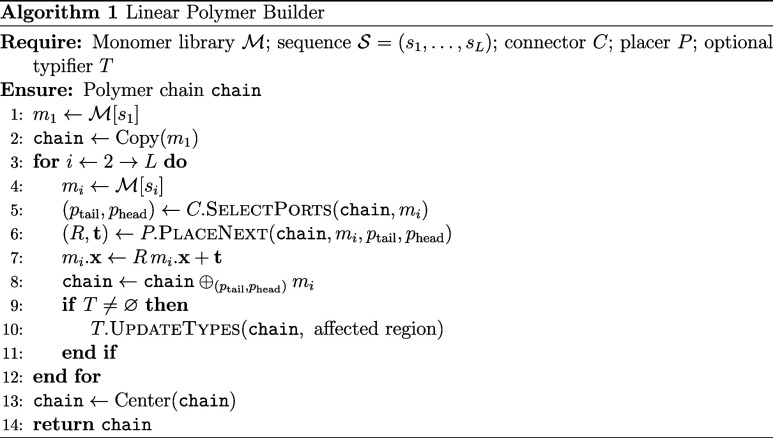


**Figure 5:**
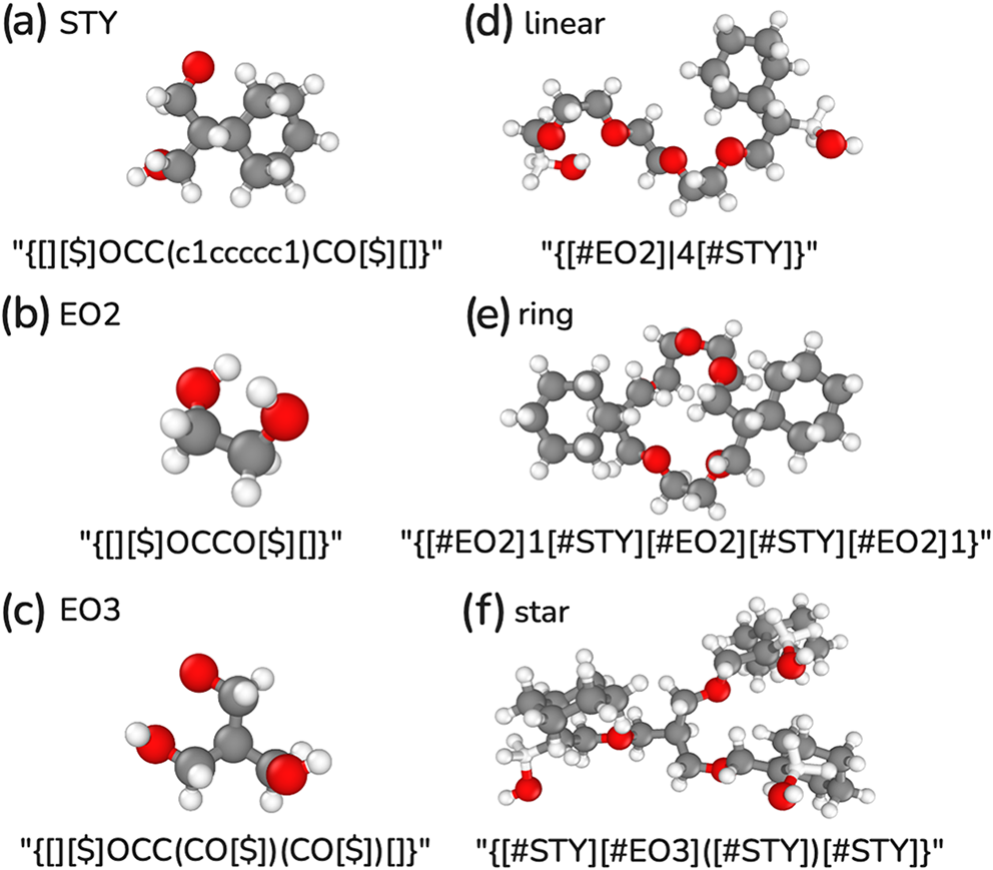
Representative building-block and architecture representations in MolPy. Panels (a–c) show atomistic building blocks for STY, EO2, and EO3, together with their corresponding string definitions. Panels (d–f) show higher-level architecture expressions constructed from these abstract building-block labels, illustrating linear, ring, and star topologies.

Polymer system construction tasks span a wide range of abstraction levels, from the sequence-driven generation of polymer ensembles to explicit modification of molecular connectivity. Despite these differences, they rely on a common set of operations on molecular structures, including defining building blocks, forming or modifying bonds, and assembling system-level configurations. MolPy adopts a modular design that factors these operations into reusable components, allowing them to be applied across different problem settings. To illustrate this, we then present two representative case studies: (iii) polymer ensemble generation from G-BigSMILES specifications and (iv) construction of reactive templates for use in molecular simulations.

The first arises from modern LLM pipelines, which motivate the use of G-BigSMILES–style representations.^46^ Traditional SMILES or BigSMILES notations describe fixed chain patterns, whereas contemporary machine-learning models can generate G-BigSMILES strings that encode reactivity probabilities, molecular-weight distributions and ensemble-level specifications in a concise declarative form. These capabilities support streamlined property prediction and automated *in silico* synthesis of polymeric materials.^1,51^ Because G-BigSMILES continues to evolve and often requires the incorporation of customized descriptors, an extensible and model-independent framework is essential for interpreting such specifications and converting them into consistent atomistic structures.

In case III, we consider a PEO-like polydisperse polymer system defined using generative BigSMILES. The corresponding BigSMILES notation defines a polymer system in which the repeat-unit connectivity and the stochastic rules governing chain growth are specified in a single declarative representation.

MolPy interprets this specification by separating structural information (repeat units and connectivity) from stochastic descriptors (e.g., chain-length distributions) through an intermediate representation (IR). Polymer sequences are generated by sampling monomers according to the specified probabilities, while chain lengths are drawn from user-defined or BigSMILES-encoded distributions (e.g., Schulz–Zimm, Poisson).

The resulting sequences are then converted into atomistic polymer chains with explicit connectivity and 3D coordinates, yielding a polydisperse ensemble of PEO-like polymer systems suitable for downstream simulation. At the system level, multiple chains are assembled to match a prescribed total mass, producing ensembles with controlled molecular weight distributions, as shown in Figure 6. The underlying algorithm is described in Algorithm 2. This example demonstrates how generative BigSMILES can be used not only to define polymer chemistry but also to construct atomistic ensembles with specified statistical properties within a unified framework.
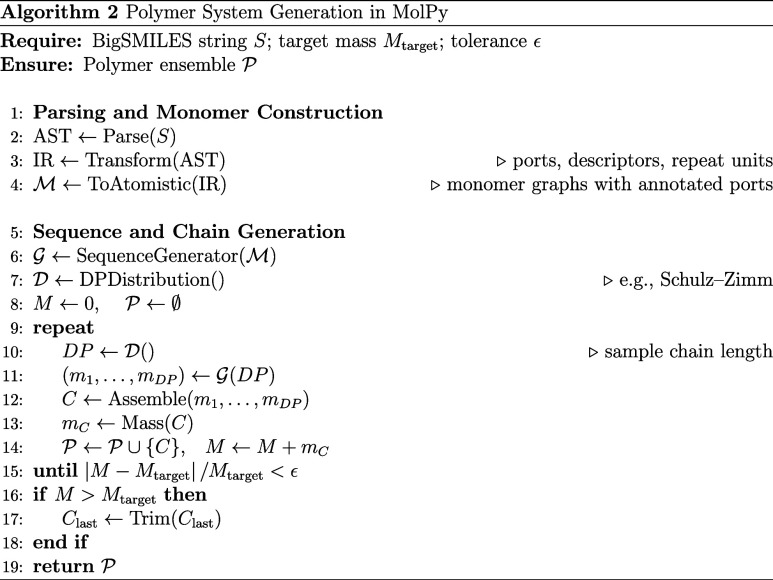


**Figure 6:**
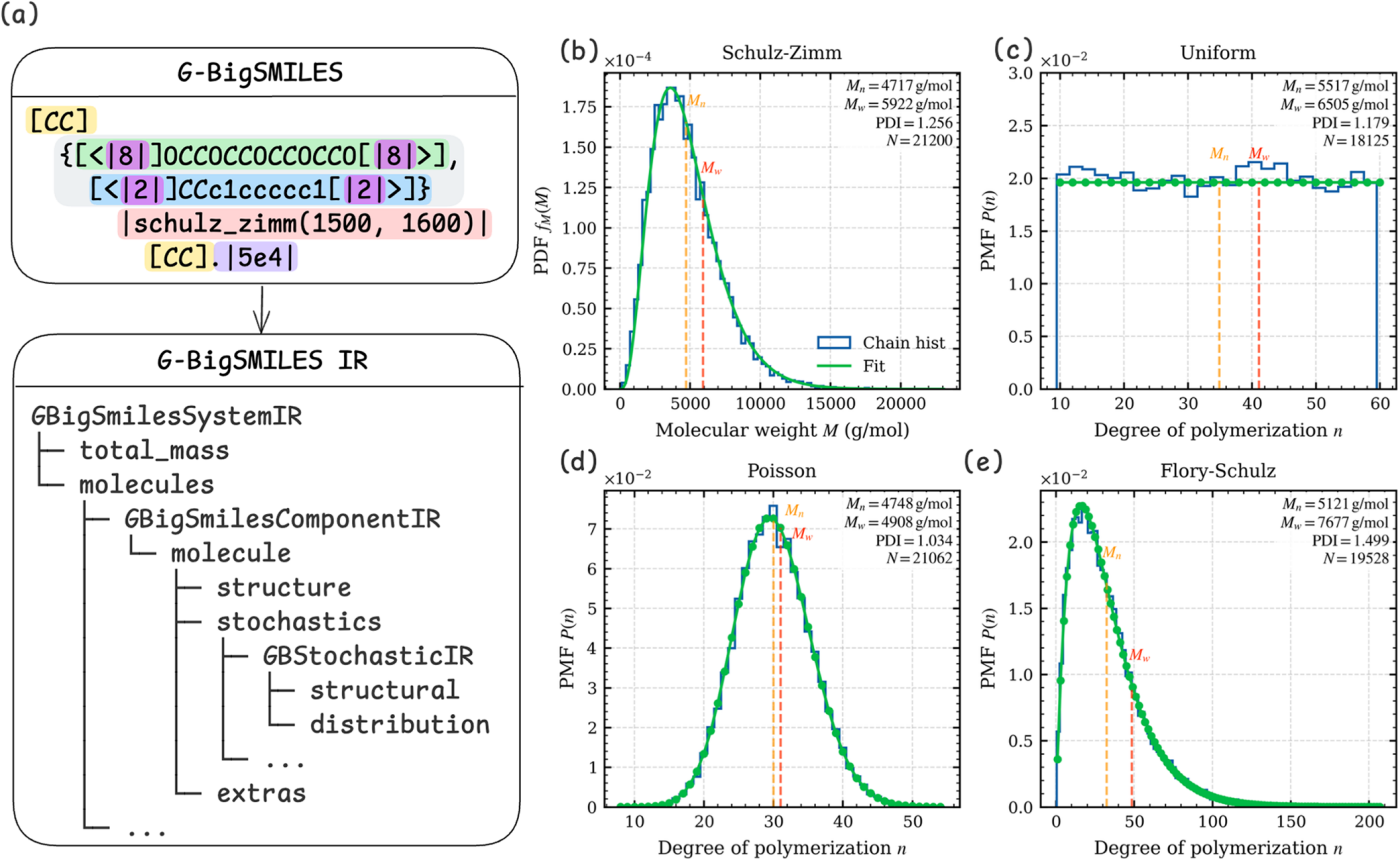
From G-BigSMILES specifications to atomistic polydisperse polymer systems via an intermediate representation (IR). (a) A G-BigSMILES expression describing a poly­(ethylene oxide) (PEO)-like polymer system, where the repeat unit connectivity and stochastic chain-length distribution (here, Schulz–Zimm) are specified in a single-declarative form. The specification is parsed into a structured G-BigSMILES intermediate representation (IR) that separates molecular structure from stochastic descriptors and distribution metadata. (b–e) Polymer ensembles generated from the IR using different degree-of-polymerization distributions: (b) Schulz–Zimm, (c) uniform, (d) Poisson, and (e) Flory–Schulz. The resulting atomistic polymer chains reproduce the target molecular-weight distributions, as shown by agreement between sampled histograms and analytical distributions (solid lines). Key metrics (*M*_*n*_, *M*_*w*_, polydispersity index PDI, and sample size *N*) are reported in each panel. The generated ensembles correspond to fully constructed atomistic polymer systems, including explicit chain connectivity and 3D coordinates, which can be directly used for downstream molecular simulations. The underlying algorithm is described in Algorithm 2.

MolPy does not attempt to provide a universal built-in solution for configuration generation; instead, it focuses on the explicit definition of how monomeric building blocks are placed, connected, and transformed within the molecular construction workflows. This design reflects the methodological diversity of modern polymer structure generation, where different tasks are often better served by specialized tools. For example, SwiftPol^15^ targets the construction and parametrization of representative polydisperse polymer systems, whereas Polyply^18^ addresses topology and coordinate generation for polymer systems under prescribed packing conditions, and PolyConf^52^ focuses on polymer conformation generation through a machine-learning approach. Rather than propose a new configuration generation method, MolPy provides reusable tools that support existing workflows or new ones without repeatedly rewriting boilerplate code.

In case IV, we apply the same modular components to a rule-based reactive simulation using bond/react-style mechanisms. Reactive simulations based on REACTER extend classical force fields with lightweight reaction rules that permit on-the-fly bond formation and breaking at a small computational cost relative to conventional reactive potentials.^45,53^ Reactions are specified through user-defined templates that describe reactant and product subgraphs, atomistic mappings, and optional geometric criteria, which may be generated programmatically rather than written manually. During simulation, candidate atom pairs are first filtered by distance, then validated by graph matching, and finally updated to the product topology, including local bonded terms and partial charges.

Although the concept of reactive topology updates is simple, preparing a complete REACTER protocol in conventional workflows often requires extensive manual editing of templates, atom mappings, retypifying rules, and topology updates across several file formats. Such a workflow is fragile, difficult to reproduce, and prone to user errors. Inspired by recent efforts by Gissinger et al.^54^ on reaction protocol generation, MolPy likewise provides components for reaction-template construction, system preparation, and file export prior to simulation. This includes selecting reaction templates from predefined reactive sites, aligning atom identifiers, forming new bonds and removing eliminated atoms, evaluating geometric feasibility, updating local topology and force field types, and generating the necessary mapping files. To illustrate this capability, the above automated protocol generation is applied in a case study to construct a cross-linked poly­(ethylene oxide) (PEO) network from two building blocks: a linear PEO trimer and a three-arm PEO junction unit.

As illustrated in Figure 7, MolPy constructs reaction templates by starting from chemically meaningful user input rather than manual template editing. Beginning with BigSMILES or annotated SMILES, MolPy first generates the corresponding atomistic structures and allows the user to specify only the reactive reference site (*init* atom) together with a topological radius that defines the spatial extent of the reaction region (Figure 7a,b). From this information, MolPy automatically identifies all atoms, bonds, angles, and dihedrals that belong to the local reaction environment, thereby defining the portion of the molecular graph that must be included in the reaction template.

**Figure 7:**
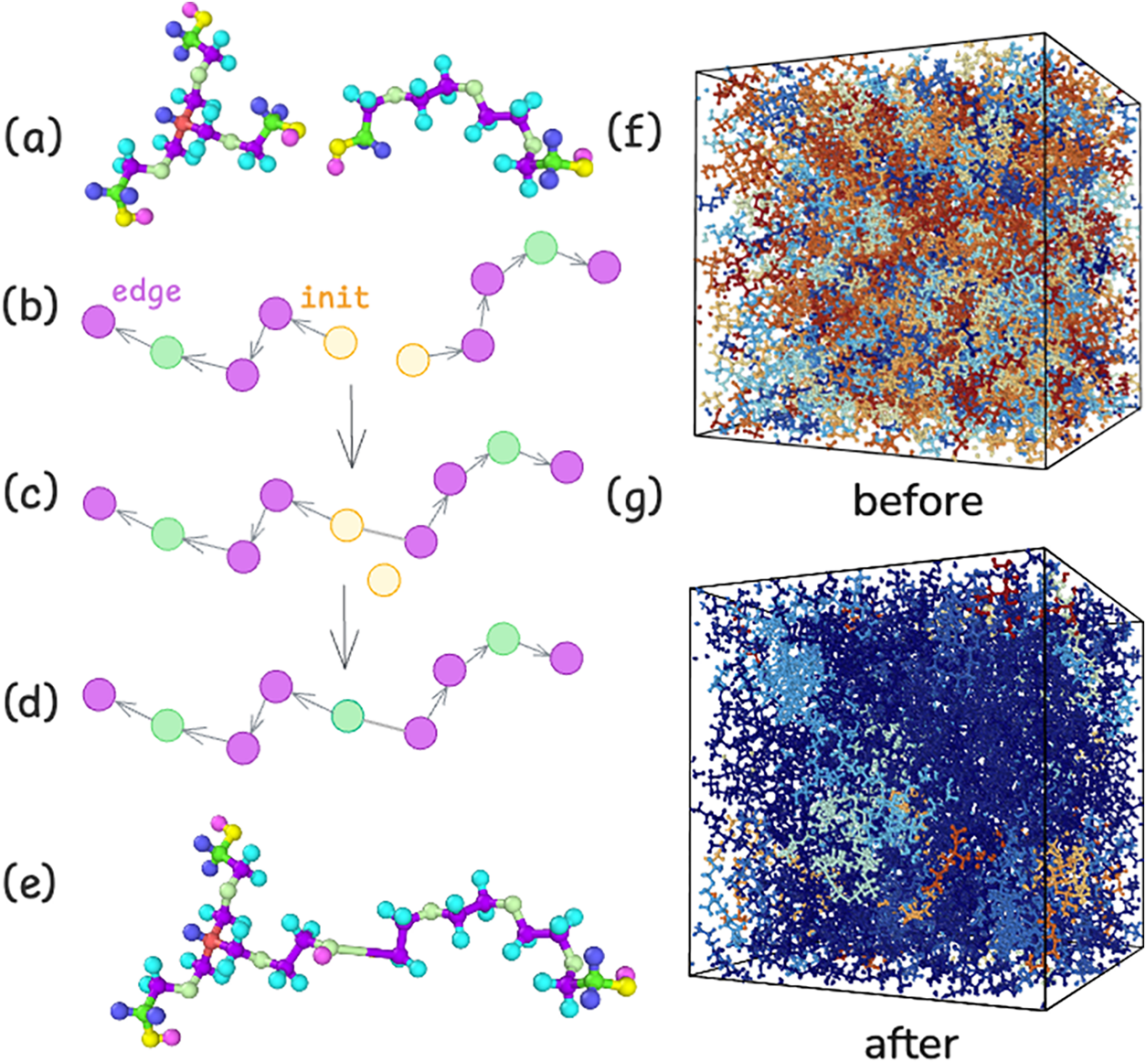
(a) Initial monomer structures before bond formation. (b–d) Schematic illustration of the topology-based template construction procedure. Starting from the user-specified initialization atoms (init), the algorithm expands the local molecular graph within a chosen topological radius to determine the spatial extent of the reaction definition until the edge atoms are reached. It then automatically generates the corresponding prereaction template, postreaction template, and atom-mapping file. (e) Resulting atomistic structure after formation of the new bond, followed by retyping of the affected atoms, bonds, angles, and dihedrals, since the local bonded environment around the newly formed bond has changed. (f-g) Representative simulation snapshots before and after the cross-linking reaction, showing the transition from the un-cross-linked system to the connected network. Colors distinguish different bond-connected molecular components.

Once this reaction region has been determined, the user-defined reaction process is applied step by step to generate the corresponding prereaction and postreaction states (Figure 7b–d). For each step, MolPy automatically constructs the paired templates together with the atom and type correspondences required between them, including the treatment of deleted atoms and the retyping of the affected local topology, as shown in Algorithm 3. In this way, template preparation is shifted from the manual editing of multiple reaction-template files to a higher-level chemical specification in which the user defines how atoms and bonds are created or removed, while MolPy automatically derives the corresponding local templates and mappings required for simulation.
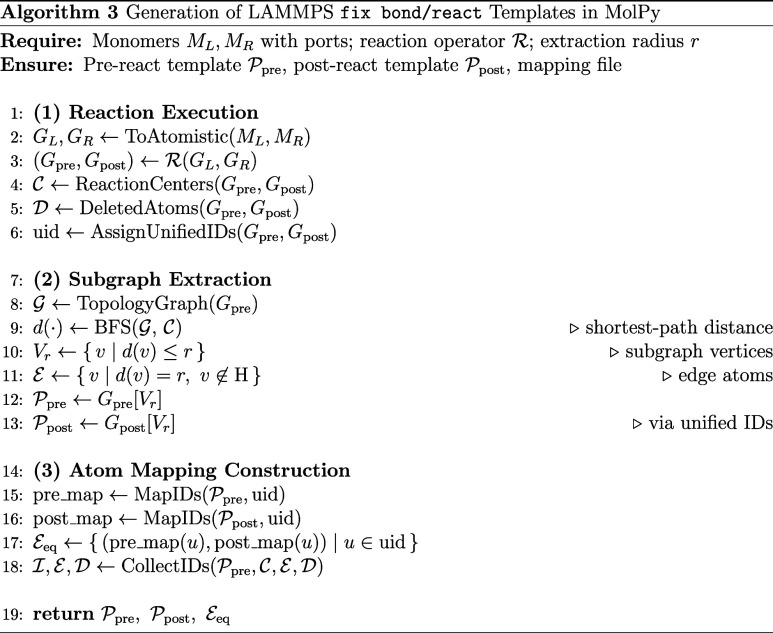


For demonstration, an initially unreacted mixture of bifunctional and trifunctional ethylene oxide monomers was first constructed at an artificially low density to facilitate coordinate generation by Packmol. The system was then compressed to a physically reasonable condensed-state density of 1.08 g cm^–3^ before reactive simulation, after which cross-linking led to the formation of a connected polymer network, as illustrated by the before/after configurations in Figure 7(f–g). Upon completion of polymerization, the final density was 1.05 g cm^–3^; the full setup used to reproduce the cross-linking-system workflow is provided in Section S1 of the Supporting Information.

Taken together, these case studies illustrate how MolPy accommodates a wide spectrum of polymer construction workflows, ranging from manual fragment assembly to probabilistic sequence generation and reactive network formation. Each scenario operates on the same underlying abstractions for structure, topology, and force field semantics, which allows new chemical rules or architectural patterns to be introduced without modifying the core framework. The progression from Case I to Case IV highlights the shift from geometry-driven procedures toward fully automated, chemistry-aware pipelines that can be composed, extended, and interpreted by both users and automated agents. By demonstrating consistent behavior across linear chains, branched topologies, and dynamically cross-linked networks, this section shows that MolPy provides a robust and extensible foundation for building polymeric systems within a unified modeling environment. The four case studies are provided as runnable examples in the User Guide of the GitHub repository and in the Supporting Information.

MolPy is built around explicit, composable data structures and transformation operators. Each component is designed to be stateless, and implicit conversions are intentionally not allowed. This design makes the intended modeling abstractions and usage patterns explicit, allowing them to be exposed through structured documentation that can be programmatically accessed by language model-based agents. In our approach, this role is fulfilled by the agent-facing MolPy documentation interface, which provides task-oriented views of MolPy documentation. This enables agents to reason about MolPy workflows without inspecting the source code and to ground code generation in documented semantics rather than heuristic pattern matching.

MolPy further provides a model context protocol (MCP) service to support efficient code retrieval and agent-assisted development through structured access to the library source code. Rather than serving as a remote execution backend, the MCP service functions as an interface for program understanding, enabling language-model agents to inspect the installed package directly instead of relying on potentially outdated or incomplete prior knowledge. Through six structured source-introspection tools, an agent can enumerate the package hierarchy, inspect exported symbols, retrieve docstrings and callable signatures, access full source implementations, and perform a codebase-wide textual search. Examples showing how prompts guide an LLM to retrieve MolPy usage examples through the MCP interface are provided in section S2 of the Supporting Information. In this way, the MCP service allows agents to obtain accurate and up-to-date information about MolPy’s API and internal organization, thereby improving the reliability of code generation, library navigation, and developer assistance.

## Vision

In this work, we introduced MolPy, an open and community-driven modeling framework, designed to unify and streamline molecular simulations. By abstracting low-level infrastructure and providing well-structured APIs and data models, MolPy enables users to focus on their scientific exploration. While full interoperability across simulation engines and file formats remains an open challenge, MolPy is architected with this long-term goal in mind and continues to evolve through a modular design, transparent development, and collaborative contributions.

To demonstrate the versatility of MolPy, we are developing a family of interoperable modules built atop its core abstractions—including AmberTools integration, molecular editing, and template generation for reactive modeling. These capabilities are already deployed in our research on self-healing polymers, polymer electrolyte design, and automated force field generation.^55^ By exposing clear, testable interfaces between modeling logic and simulation backends, MolPy provides a foundation for incorporating machine learning, domain-specific languages, and interactive visualization directly into the simulation workflow.

Looking ahead, MolPy is intentionally designed to be anLLM-friendly. As agents increasingly participate in workflow composition, simulation setup, and data analysis, modeling frameworks must provide consistent abstractions, machine-readable schemes, and predictable operator semantics. MolPy’s explicit data models, uniform transformation rules, and modular operators allow intelligent agents to compose pipelines, generate or validate inputs, and automate simulation tasks with a minimal amount of human intervention.

MolPy’s explicit data structures, deterministic transformation rules, and modular operators provide a suitable foundation for reactive topology editing in polymer simulations, enabling agents to assemble workflows, validate inputs, and automate repetitive modeling tasks in a controlled and reproducible manner. In this sense, MolPy serves as a flexible and extensible framework that supports both human-driven and agent-assisted molecular-modeling workflows.

## Supplementary Material





## Data Availability

The code, tutorials, and runnable examples used in this study are publicly available in the MolPy repository at https://github.com/MolCrafts/molpy. Supporting methodological details, including the cross-linking simulation procedure and the AI agent providers, setup, and prompts, are provided in the Supporting Information. The data underlying this study are therefore available in the article, its Supporting Information, the public repository cited above, and the files submitted together with the manuscript. Runnable stepwise tutorials corresponding to the case studies discussed in this article are provided in the repository and in the Supporting Information.

## Notes

The system was then equilibrated in two stages: first under the NVT ensemble at 300 K for 500 ps, and subsequently under the NPT ensemble at 300 K and 1 atm for an additional 50 ns to allow density relaxation (PDF)
Output files for all study cases, including representative generated structures, intermediate and final outputs, logs, and other files needed to inspect the results reported in the manuscript (ZIP)
