# Supplementary material for: MolPy: A Large Language Model-Friendly Toolkit for Reactive Topology Editing in Polymer Simulations
Source: J Chem Inf Model. 2026 Jul 1;66(13):7562–72. doi: 10.1021/acs.jcim.6c01137 (PMC13370848; doi:10.1021/acs.jcim.6c01137)
Supplement: Supplementary file 1 [file ci6c01137_si_001.pdf]

MolPy: a Large Language Model-friendly toolkit for reactive topology editing in polymer simulations

Jichen Li,[*,†] Fabian Schwarz,[†] Wentao Guo,[‡] Ge Sun,[¶,§] and Daniel Brandell[*,†]

†*Department of Chemistry-Ångström Laboratory, Uppsala University, 75121 Uppsala, Sweden*

‡*Division of Chemistry and Chemical Engineering, California Institute of Technology, Pasadena, CA 91125, United States*

¶*Courant Institute School of Mathematics, Computing, and Data Science, New York University, New York, NY 10012, United States*

§*Department of Chemical and Biological Engineering, Tandon School of Engineering, New York University, Brooklyn, New York 11201, United States*

E-mail: jichen.li@kemi.uu.se; daniel.brandell@kemi.uu.se

Supporting Information Available

S1. Reproducibility of Crosslinking system

Crosslinked polymer networks were constructed using two hydroxyl-terminated monomer species, denoted `EO2` and `EO3`, which represent bifunctional and trifunctional ethylene oxide building blocks, respectively. Monomer structures were first parsed from their string-based representations and converted into atomistic three-dimensional geometries with explicit hydrogens. Molecular topology, including bonds, angles, and dihedrals, was then generated,

after which all atoms were assigned OPLS-AA force-field types using the MolPy typification workflow. Atom identifiers were assigned prior to force-field typing to ensure compatibility with subsequent LAMMPS export.

Reactive templates for LAMMPS `fix bond/react` were generated using the `BondReactReacter` workflow implemented in MolPy. Template generation was based on a dehydration-condensation reaction in which a bond is formed between two reactive sites while the corresponding leaving groups are removed. In the present system, EO2 and EO3 share the same local reactive arm chemistry, such that a single reaction template was sufficient despite the presence of two distinct monomer species. This follows from the fact that `fix bond/react` matches templates according to the local bond topology around the reaction center rather than whole-molecule identity. A graph radius of four bond hops was used for subgraph extraction, such that the pre-reaction and post-reaction templates captured the complete local chemical environment of the reactive site while keeping all atoms with modified force-field types sufficiently far from the template boundary. The resulting template set consisted of pre-reaction and post-reaction molecular fragments together with the associated atom mapping and deletion information required by LAMMPS.

Initial configurations were generated by packing EO2 and EO3 monomers into a cubic simulation box using Molpack, which wraps Packmol for non-overlapping coordinate generation. In the example shown here, 600 EO2 molecules and 200 EO3 molecules were used. The box length was determined from the total molecular mass and a target density of 0.3 g cm^{-3}, with the low initial density chosen to facilitate Packmol packing. The packed configuration was then assigned periodic boundary conditions and exported together with the reaction templates and the complete force-field definition using a unified type-mapping scheme. This ensured that atom, bond, angle, and dihedral type indices remained consistent across the initial configuration, the force-field parameter file, and the bond/react templates.

Reactive molecular dynamics simulations were carried out using LAMMPS(4 Feb 2025). Long-range electrostatic interactions were treated using PPPM with an accuracy of 10^{-4}, and

bonded exclusions were handled using `special_bonds` settings appropriate for the chosen OPLS-AA force field. The packed configuration was first relaxed by conjugate-gradient energy minimization to remove steric clashes introduced during packing. The system was then equilibrated in two stages: first under the NVT ensemble at 300 K for 500 ps, and subsequently under the NPT ensemble at 300 K and 1 atm for an additional 50K ps to allow density relaxation. Crosslinking was then activated using `fix bond/react`. During this stage, newly reacted atoms were temporarily assigned to a separate thermostat group to stabilize the local structure immediately after bond formation. Reactive MD was performed for 500 ps under periodic boundary conditions, while atomic coordinates and bond formation events were recorded throughout the simulation.

At the end of the reactive simulation, molecule identifiers were recalculated from the final bond topology, and the final configuration was written to disk for analysis. Successful crosslinking was assessed by comparing the number of connected molecular components before and after the reaction run. A decrease in the number of molecular entities relative to the initial system indicated the formation of intermolecular crosslinks and the emergence of network structure.

S2. Reproducibility of LLM-assisted code generation

To make the LLM-assisted examples in this work fully transparent and reproducible, we document the complete generation conditions for each case study in the Supporting Information. For every example, we provide the exact user prompt, the generated code, and the corresponding generation environment. This includes the model used for generation, the availability of structured tool access through the built-in MolPy MCP (Model Context Protocol) server, and the fact that package exploration was performed through explicit tool calls rather than undisclosed contextual injection.

The example were generated in clean sessions, without hidden prior conversation state or manually supplied background context beyond the prompt itself and the functionality

explicitly exposed through MCP. This ensures that the reported outputs reflect an auditable, tool-mediated generation process rather than reliance on opaque or irreproducible context.

Model and environment. Code generation was carried out using Claude Sonnet 4.6 in Claude Code with access to the MolPy MCP server. The MCP server exposed structured, tool-based interfaces to the MolPy package, including package navigation, module inspection, and function lookup. All examples were generated with MolPy version 0.3.0 in clean sessions, without undisclosed prior context or manual context injection. AmberTools was additionally required, and installation instructions are available from its official website. The generation prompts and resulting scripts are provided herein, and the complete model outputs together with the corresponding tool interactions are included in the accompanying data.

Prompt

```
Use MolPy to generate an atomistic  PEO/LiTFSI polymer electrolyte system
with the following strict constraints. Build  polydisperse PEO chains  using
a  SchulzZimm distribution  with a target  number-average degree of
polymerization (DP_n = 20)  and  polydispersity index (PDI =
1.20). Construct exactly  40 PEO chains, and choose the SchulzZimm
parameters so that the sampled ensemble matches these targets as closely as
possible; report the resulting (M_n), (M_w), PDI, and the full chain-length
distribution. Use  AmberTools  to generate the force-field
parameters and connectivity for the PEO monomer and polymer chains, using GAFF
with chemically correct linkage and end-group handling. Add LiTFSI  salt at
a fixed composition of  EO:Li = 20:1 , and compute the exact number of
LiTFSI molecules from the total number of EO repeat unitsin the sampled
polymer ensemble. Look up literatures for Li+ nonbond
parameters. Build the final mixed system by combining the polydisperse PEO
chains with LiTFSI and then packing them using  Packmol . Because the initial
```

```
 structure is only intended as a loose starting configuration before
equilibration, use a very low initial density of 0.10 g/cm(ˆ3)  and size the
 simulation box accordingly. The workflow should be fully end-to-end and
clearly structured: define the PEO repeat unit and LiTFSI, sample chain
lengths from the SchulzZimm distribution, build all PEO chains,
assign parameters and topology with AmberTools, add the required number of
LiTFSI molecules, generate a Packmol input file, pack the full system, and
export the resulting coordinates and topology/force-field files for downstream
molecular dynamics. Please provide a brief summary of the
construction strategy, then the complete runnable code, and finally a concise
report of the generated system including the chain count, sampled
chain-length statistics, total EO units, LiTFSI count, EO:Li ratio, box size,
and initial density. If any MolPy interface is missing, make the most
reasonable implementation based on the existing API and clearly state the
assumptions.
```
